# “A basic frame existed but was not followed” – Finnish public health system's response to COVID-19: Moona Huhtakangas

**DOI:** 10.1093/eurpub/ckac131.009

**Published:** 2022-10-25

**Authors:** M Huhtakangas, L Kihlström, S Karreinen, I Keskimäki, L-K Tynkkynen

**Affiliations:** 1 Welfare State Research and Reform, Finnish Institute for Health and Welfare, Helsinki, Finland; 2 Health Sciences, Faculty of Social Sciences, Tampere University, Tampere, Finland

## Abstract

**Background:**

Crisis management Managing crises often requires diverging from predetermined plans. In this paper, we investigate how public health authorities in Finland acted, what kind of roles they adopted and how the expected roles and actions appeared in relation to the legislative framework and preparedness plans during the COVID-19 pandemic. Based on inter-country comparisons, Finland has managed COVID-19 pandemic relatively well. The study provides qualitative insights on pandemic governance in a decentralized multi-stakeholder public health system.

**Methods:**

Semi-structured interviews (n = 53) with key public health actors at central, regional and local levels were conducted during March 2021-February 2022. The data was analysed with thematic analysis.

**Results:**

The predetermined roles and duties for pandemic management were not unequivocal in practice and appeared unrealistic considering the resources of the public health system. Responsibility was divided between several actors, but lack of interaction enhanced emerging tensions between them. Local and regional actors experienced national steering intervening in operational decisions. At central level distrust towards the capabilities of local and regional actors was expressed. The pandemic was framed and managed as a health crisis despite of its wider societal effects. This challenged local and regional decision-making, where wider societal impacts had to be considered.

**Conclusions:**

Public health authorities in Finland interpreted their roles and responsibilities in pandemic governance in various ways: some actors adopted more active agency than others and the roles were not always in line with the existing regulative framework.

**Key messages:**

• Interpretation of the roles outlined in preparedness plans are context dependent and may lead to conflicts between different actors.

• In a system with multiple actors at multiple levels, building trust and improving interaction are important for coordinated action.

